# Transcriptomic and Metabolomic Insights into Age-Related Changes in Lung Tissue of Yaks Under Highland Stress

**DOI:** 10.3390/ijms252212071

**Published:** 2024-11-10

**Authors:** Changze Cui, Shaopeng Chen, Baohong Mi, Youpeng Qi, Chenyue Jiao, Meixian Zhang, Yiduo Dai, Xiangyan Wang, Jiang Hu, Bingang Shi, Jiqing Wang, Zhidong Zhao, Xiu Liu, Xiaolan Zhang

**Affiliations:** Gansu Key Laboratory of Herbivorous Animal Biotechnology, College of Animal Science and Technology, Gansu Agricultural University, Lanzhou 730070, China; 107332202075@st.gsau.edu.cn (C.C.); 15095373670@163.com (S.C.); mi_baohong@163.com (B.M.); qiyp_gsau@163.com (Y.Q.); 1073323120227@st.gsau.edu.cn (C.J.); zmx15719616201@163.com (M.Z.); 107332212162@st.gsau.edu.cn (Y.D.); wxy9242022@163.com (X.W.); wangjq@gsau.edu.cn (J.W.); zhaozd@gsau.edu.cn (Z.Z.); liuxiu@gsau.edu.cn (X.L.); zhangxl8997@163.com (X.Z.)

**Keywords:** yak, lung, high-altitude hypoxia, age-related

## Abstract

As an indigenous species on the Tibetan Plateau, the yak is well adapted to the plateau hypoxic environment. The high-altitude hypoxia adaptation of the yak requires the adaptive reshaping of multiple tissues and organs, especially the lungs. To reveal the adaptive development of yak lungs under hypoxic stress at the tissue and molecular levels, we conducted histomorphological observations as well as transcriptomic and metabolomic studies of yak lungs at three ages (0.5, 2.5, and 4.5 years). The results showed that the lung tissue developed significantly with age. The mean alveolar area was higher (*p <* 0.01) in 4.5 and 2.5-year-old yaks than in 0.5-year-old yaks. The percentage of elastic fibers, micro-arterial wall thickness, and micro-arterial area showed an increasing trend (*p <* 0.01) from 0.5-year-old yaks to 2.5-year-old yaks and then to 4.5-year-old yaks. In addition, some critical differentially expressed genes related to angiogenesis (*MYC*, *EPHA2*, *TNF*), fiber formation (*EREG*), smooth muscle proliferation (*HBEGF*), erythropoiesis (*SOCS3*), and hypoxia response (*ZFP36*) were identified. Some metabolites associated with these genes were also found simultaneously. These findings provide a deeper understanding of the molecular strategies underlying this species’ extraordinary ability to survive normally in low-oxygen environments. In conclusion, the lungs of yaks undergo continuous adaptive development under hypoxic stress, and these findings are crucial for understanding the molecular mechanisms by which native species of the Tibetan Plateau survive in harsh environments.

## 1. Introduction

The Tibetan Plateau, the highest plateau on earth, with an average elevation of more than 4000 m [1] and covering one quarter of China’s land area, is known as the “Roof of the World” and the “Third Pole”. The partial pressure of oxygen decreases with increasing altitude. At 2800 m, pO2 is only 70% of sea level [2], which leads to hypoxia in animals and poses a severe test for native species on the Tibetan Plateau. Therefore, the Tibetan Plateau can be used as a natural laboratory to study the adaptation of organisms to plateau hypoxia. The Yak is the only bovine species that can grow and reproduce on the Tibetan Plateau, and they are known as the “boat of the plateau” and “all-purpose livestock” [3] because they can withstand the harsh environmental conditions such as hypoxia and cold and the short growth cycle of pasture. After a long period of evolution by natural and artificial selection, yaks have gradually developed unique physiological and molecular mechanisms to adapt to the plateau hypoxic environment, making them an ideal animal model.

As the most critical organ in the respiratory system, the lung is an essential functional organ for plateau animals to adapt to the low-oxygen environment. It has recently attracted extensive attention from scholars at home and abroad. In a study on low-oxygen adaptation in Tibetan sheep at different altitudes, it was found that the area and number of alveoli in the lungs of Tibetan sheep were positively correlated with the altitude; in addition, the air–blood barrier was also gradually thinned with the increase in altitude [4]. To have higher pulmonary vascular volume, the number of pulmonary micro-arterioles in Tibetan sheep at higher altitudes was more numerous, and their area was also more significant. When the cardiac output of Tibetan sheep increases with the rise in altitude, to avoid pulmonary hypertension, the pulmonary arteries would need to have a solid diastolic and contractile capacity, and the thickness and elastic fiber content of the pulmonary artery wall increases with altitude [5]. These changes were found simultaneously in studies of hypoxic acclimatization in rats [6]. In addition, plateau animals’ blood physiological and biochemical indices undergo corresponding adaptive changes. These features enable plateau animals to enable the organism better to exchange gases with the outside world and to better adapt to the hypoxic environment [7,8,9,10,11].

In order to have a larger space for the development of the heart and lungs, yaks have more extended ribs and larger rib spacing, which can expand the chest volume. As a percentage of the carcass, yak lungs also increase gradually with increased elevation. At the same time, the larger diameter of the trachea in yaks can also increase the volume of air entering the body [12]. Comparing the histomorphology of the lungs of yaks with that of cattle, the alveolar septa were thinner in yaks than in cattle, and the thickness of the smooth muscle of the fine bronchi at all levels was more significant [13]. The elastic fiber content of yak alveolar tissue increases significantly after birth, improving oxygen utilization efficiency in the plateau environment [14]. In recent years, transcriptomics has been applied to the study of hypoxic adaptation in plateau animals [12,14,15,16,17,18]. These studies are all centered around different altitudes to explore the hypoxic adaptation of plateau animals. Their associated adaptive changes are not linearly related to altitude, suggesting that hypoxic adaptation in plateau animals does not rely on changes in a single or specific trait but rather on a series of complex adaptive mechanisms for different species. Researchers often overlook nutritional status in studies of the hypoxic acclimatization of highland animals at different altitudes regardless of whether they are housed or grazed. However, nutritional status often plays a crucial role in animal development.

Previous studies found that energy in the lungs is normally used to carry out cellular activities such as gene transcription, protein translation [19], DNA replication [20], and repair [21]. However, the lungs contain several highly specialized cell populations that engage in more unique forms of energy-consuming behaviors such as airway clearance (phagocytosis and ciliary motility) [22], bronchial gland secretion, airway and vascular constriction, and the production of lung surfactant. Energy expenditure within the lungs is critical not only for regulating general cellular functions but also for maintaining the unique activities of the organ. Lung metabolic function is often overlooked in studies of animal adaptation to the low-oxygen environment of the plateau.

In order to gain a comprehensive understanding of the age-specific changes in lung tissue of yaks under plateau stress, yaks aged 0.5 years (calves), 2.5 years (young cows), and 4.5 years (adult cows) were selected in the present study. Lung structures were observed using light microscopy, and genes and metabolites of lung tissues were quantitatively analyzed using the multi-omics techniques of transcriptomics and metabolomics. This work led to a better understanding of age-specific changes in lung tissue morphology, gene expression, and metabolic patterns in yaks under plateau stress.

## 2. Results

### 2.1. Physiological Differences in the Lungs

**Figure 1:**
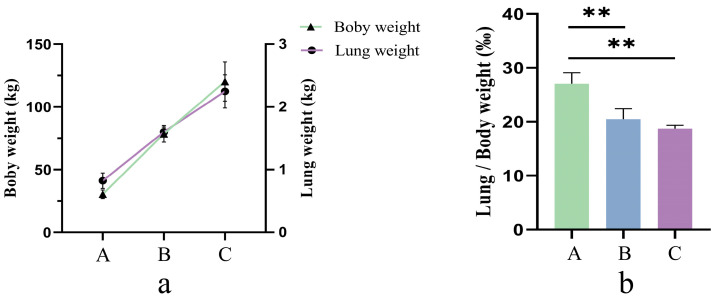
(**a**) Yak body weights and lung weights; (**b**) differences in lung as a percentage of body weight. A: 0.5-year-old yaks, B: 2.5-year-old yaks; C: 4.5-year-old yaks. ** *p <* 0.01.

The lung weight/carcass weight ratio showed a gradual decrease with age, and that at 0.5 years old was lower than that at 2.5 years old (*p* < 0.01) (Figure 1).

**Figure 2:**
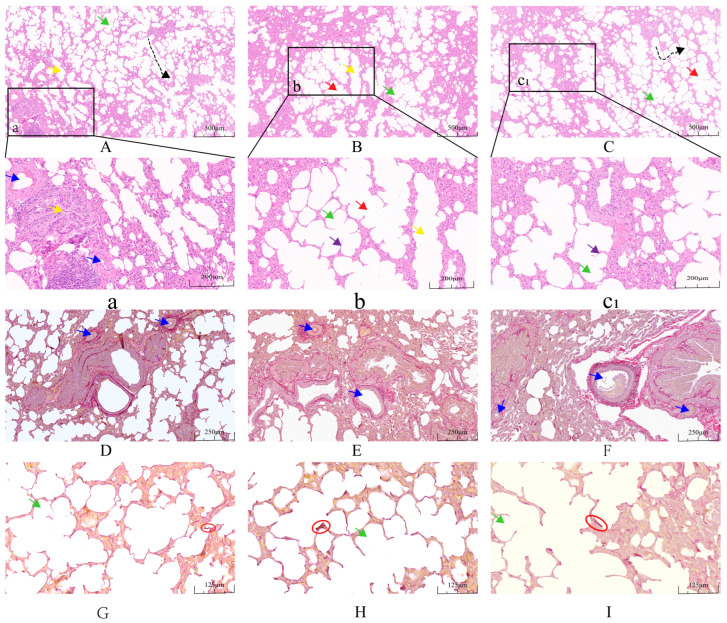
The lung hematoxylin–eosin (HE) staining and Weigert resorcinol magenta (WRM) staining. Figures (**A**–**C**) show the differences in HE sections of lungs from 0.5, 2.5 and 4.5-year-old yaks at 10× field of view. Figures (**a**), (**b**) and (**c1**) show magnified images of the black boxes in (**A**), (**B**) and (**C**), respectively. Figures (**D**–**F**) show the differences between HE sections of lungs from 0.5, 2.5 and 4.5-year-old yaks at 20× field of view. Figures (**G**–**I**) show the differences in WRM sections of lungs from 0.5, 2.5 and 4.5-year-old yaks at 40× field of view. Green arrows indicate alveoli, black arrows are in the path of alveolar ducts, yellow arrows indicate terminal fine bronchi, blue arrows indicate arterioles, purple arrows indicate alveolar septa, red circles represent elastic fibers, and red arrows indicate respiratory fine bronchi.

The lung tissue morphology of yaks at different ages is shown in Table 1 and Figure 2. The number of alveoli in 0.5-year-old yaks was higher than that in 2.5-year-old yaks (*p <* 0.01), and that in 2.5-year-old yaks was higher than that in 4.5-year-old yaks (*p <* 0.01). The mean alveolar surface of 4.5 and 2.5-year-old yaks was higher than that of 0.5-year-old yaks (*p <* 0.01). The lung elastic fiber ratio, arteriole wall thickness, and arteriole surface of 4.5-year-old yaks were higher than those of 2.5-year-old yaks (*p <* 0.01), and those of 2.5-year-old yaks were higher than those of 0.5-year-old yaks (*p <* 0.01).

### 2.2. The Effect of Different Ages on Gene Expression in Lung Tissue

Next, an attempt was made to study the effect of different ages on gene expression in lung tissues. Fifteen independent cDNA libraries were constructed from lung tissues of yaks (five each for ages A, B, and C). These cDNA libraries were used for RNA-seq, and the RNA-seq data are summarized in Table 2. On average, ages A, B, and C produced 44,770,822, 45,910,218, and 43,345,683 raw reads, respectively. After filtering the reads containing adapters, an average of 44,258,157, 45,422,947, and 42,860,926 clean reads were obtained from the three ages, respectively. The average useful reads rate was 98.88%, 98.94%, and 98.88%. Of the remaining clean sequences, an average of 41,661,962 (94.13%), 42,744,048 (94.10%), and 40,212,112 (93.81%) matched well with the yak genome assembly (Bos_grunniens.LU_Bosgru_v3.0.dna.toplevel.fa) with 90.78%, 90.72% and 90.79% unique matches, respectively.

When comparing A vs. B, A vs. C, and B vs. C, 342, 184, and 207 genes were identified as differentially expressed (FDR < 0.05, fold change ≥ 2 or ≤0.5), respectively. Of these, 98, 85, and 130 genes were significantly up-regulated, and 244, 99, and 77 were significantly down-regulated.

**Figure 3:**
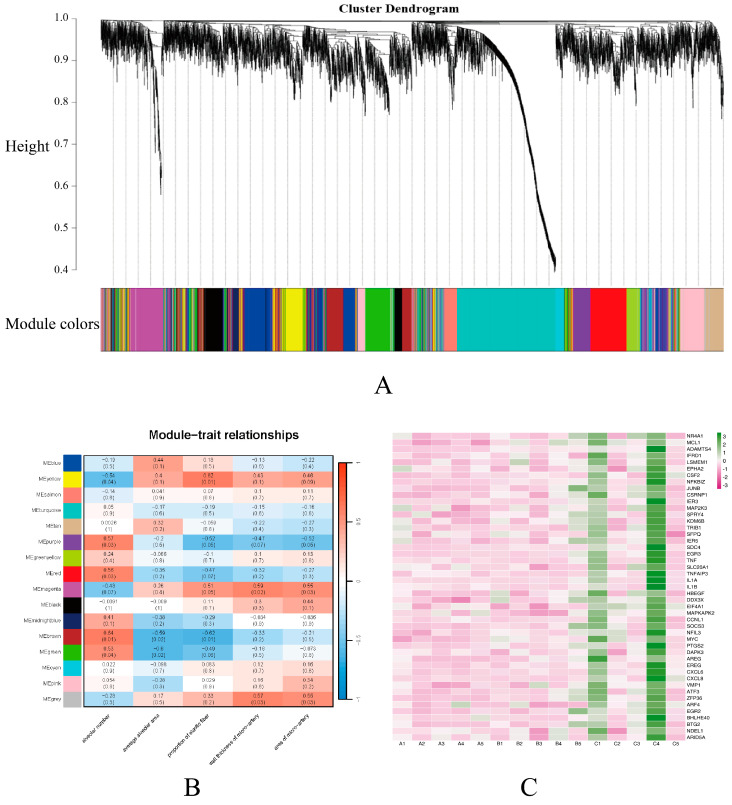
Gene co-expression networks and their relationship with phenotype in yak. (**A**) Dendrograms from gene co-expression networks of samples in three groups (A, B and C). (**B**) Relationship of alveolar number, mean alveolar area and elastic fiber percentage with 16 gene modules (MEblue to MEgray). Blue color indicates negative correlation while red color indicates positive correlation. Values within the squares indicate Pearson correlation r-values and *p*-values. *p*-values are in parentheses at the end. (**C**) Heat map showing gene expression in the MEmagenta module. The color of cells from green to red corresponds to low to high relative gene expression. Text on the right side of the graph indicates the gene name, while the group name and sample ID are labeled on the bottom side of the graph.

Weighted gene co-expression network analysis (WGCNA) was used to screen gene modules to determine the effect of factors such as lung histomorphology and age on the integrated expression of genes. As a result, 16 modules of co-expressed genes (named colors, MEblue–MEgray, respectively) were identified (Figure 3A,B). The MEmagenta module showed the strongest correlation with an alveolar number, mean alveolar area, and elastic fiber percentage, and its central genes are shown in the heat map (Figure 3C). Among them, MEmagenta had the lowest expression abundance in group C.

**Figure 4:**
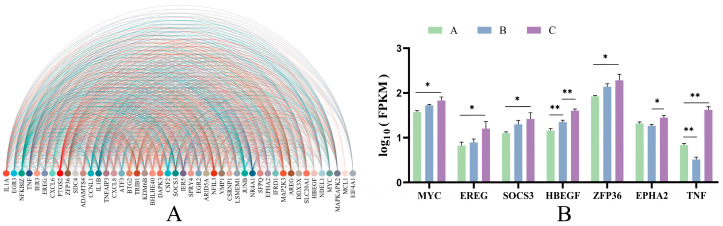
Interaction graph and relative expressions of the hub genes in the MEmagenta module. (**A**) Network analysis of the hub genes. (**B**) The relative expressions of 7 genes (including *MYC*, *EREG*, *SOCS3*, *HBEGF*, *ZFP36*, *EPHA2* and *TNF*) based on transcriptomics are shown by histograms. A: 0.5-year-old yaks, B: 2.5-year-old yaks; C: 4.5-year-old yaks. * 0.01 < *p <* 0.05, ** *p <* 0.01.

Interaction arc diagrams were made for the significant genes (Figure 4A), which were arranged by the number of connections from left to right. The primary functions are related to growth and development and hypoxia regulation, including animal organ development (GO:0048513), blood vessel endothelial cell migration (GO:0043534), regulation of smooth muscle cell proliferation (GO:0048660), blood vessel development (GO:0001568), angiogenesis (GO:0001525), regulation of hemopoiesis (GO:1903706), smooth muscle cell proliferation (GO:0048659), positive regulation of fibroblast proliferation (GO:0048146), cellular response to decreased oxygen levels (GO:0036294), and cellular response to hypoxia (GO:0071456). The central genes annotated in the above functions are MYC Proto-Oncogene (*MYC*), Epiregulin (*EREG*), Tumor Necrosis Factor (*TNF*), Heparin Binding EGF-Like Growth Factor (*HBEGF*), ZFP36 Ring Finger Protein (*ZFP36*), EPH Receptor A2 (*ZPHA2*), and Suppressor of Cytokine Signaling 3(*SOCS3*). As expected, five genes (*MYC*, *EREG*, *SOCS3*, *HBEGF*, and *ZFP36*) increased significantly (*p <* 0.05) with age (Figure 4B).

### 2.3. Validation of RNA-Seq Results by RT-qPCR

**Figure 5:**
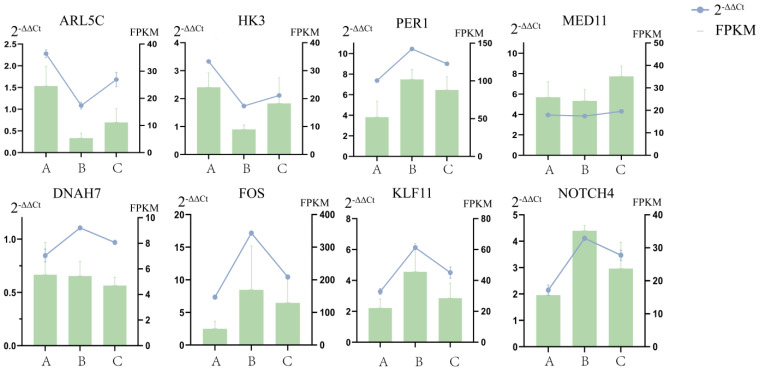
Comparison of gene expression levels measured using RNA-seq and RT-qPCR for 8 randomly selected DEGs. 2^−ΔΔCt^: The relative expression of a gene when a sample is subjected to RT-qPCR.

Eight DEGs were randomly selected for RT-qPCR, and the results were consistent with the RNA-seq data (Figure 5). This indicates that the quantitative analysis of gene expression in yak lungs by RNA-seq is reliable and repeatable.

### 2.4. The Metabolites in Lung Tissue Influenced by Different Age

**Figure 6:**
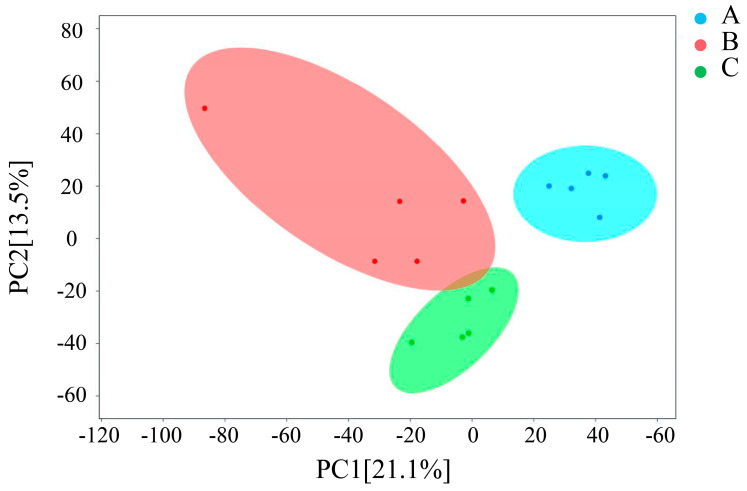
The principal component analysis (PCA) of metabolite composition in different groups (A, B and C). Each point represents a unique sample, and different colors represent different groups.

Metabolomics techniques were used to detect the effect of the same age on lung tissue metabolites. A total of 814 metabolites were detected in 15 samples by passing through the LC-MS instrument. Principal component analysis (PCA) showed significant differences in metabolite composition between groups (Figure 6).

**Figure 7:**
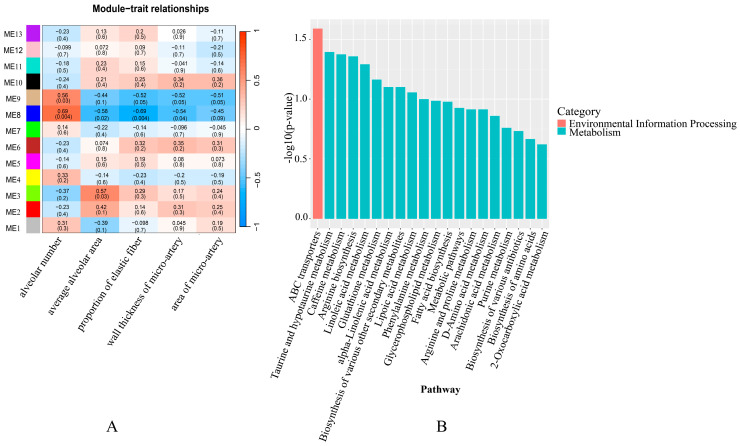
The functions of key metabolites associated with different ages. (**A**) Relationship between alveolar number, mean alveolar area, elastin fiber percentage and the 13 metabolite modules (ME1 to ME13) calculated by WGCNA. (**B**) Enrichment analysis of the metabolites in the ME2 module.

Furthermore, WGCNA was performed to cluster metabolites and identify modules associated with lung tissue morphology. As a result, 13 modules (ME1 to ME13) were classified (Figure 7A). The ME2 module correlated strongly with an alveolar number, mean alveolar area, and elastic fiber percentage. Enrichment analysis was then used to identify pathways for 75 metabolites in the ME2 module. The metabolic pathways described were mainly enriched for linoleic acid metabolism, glycerophospholipid metabolism, arginine and proline metabolism, D-amino acid metabolism, arachidonic acid metabolism, D-amino acid metabolism, arachidonic acid metabolism, and other metabolic pathways (Figure 7B).

### 2.5. Correlation of Hub Genes of MEmagenta Module with Metabolites of ME2 Module

**Figure 8:**
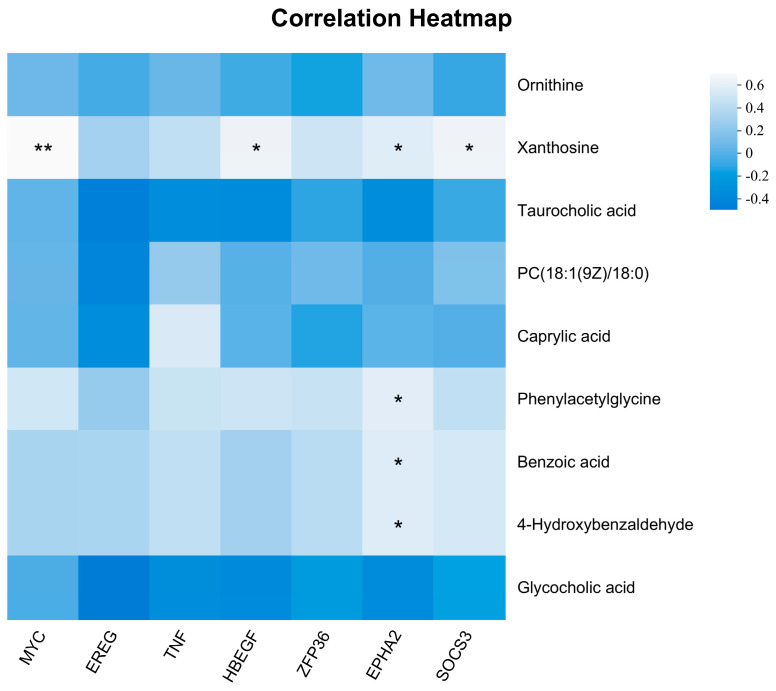
Spearman’s correlation with MEmagenta’s hub genes and metabolites in the ME3 module. * 0.01 < *p <* 0.05, ** *p <* 0.01.

Correlations were calculated between the hub genes of the MEmagenta module and all metabolites of the ME2 module (Figure 8). The *MYC*, *HBEGF*, *EPHA2*, and *SOCS3* hub genes correlated highly with some metabolites. For example, *MYC* was highly significantly positively correlated with xanthosine (*p <* 0.01), and *HBEGF* and *SOCS3* were significantly positively correlated with xanthosine (*p <* 0.05). *EPHA2* was positively correlated with xanthosine, while phenylacetylglycine, benzoic acid, and 4-hydroxybenzaldehyde were significantly positively correlated (*p <* 0.05).

## 3. Discussion

### 3.1. Adaptive Changes in Lung Histomorphometry

The lung weight ratio to carcass weight in yaks may be related to their gradual postnatal acclimatization to a low-oxygen environment. Previous studies have shown that the proportion of lung weight to carcass weight in Tibetan sheep increases gradually with altitude [4] and that the lung capacity of Quechua women at high altitudes is more significant than that of women on the plains [23]. In the present study, five calves, young and adult yaks with similar body conditions and good health, were selected, and their carcass weights and lung wet weights were weighed. It was found that the lung weight ratio of yaks gradually decreased with age, and the lung weight ratio of 0.5-year-old yaks was highly significantly higher than that of 2.5- and 4.5-year-old yaks. Previous studies on hypoxic adaptation in highland animals have set the study subjects at different altitude prerequisites and found that larger lungs and high lung ventilation may result from adaptation to the hypoxic environment in high-altitude animals [24]. The present study selected yaks at different growth stages at the same altitude. It was found that a larger lung-to-weight ratio could support calves’ adaptation to hypoxia when they had not yet developed a more complete lung microstructure. In contrast, young and adult yaks had more developed lungs and did not need a larger lung-to-weight ratio to adapt to hypoxia. This phenomenon seems to differ from the performance of Tibetan sheep adapted to the lung-to-weight ratio in a low-oxygen environment. This may be because during growth, yaks are mainly focused on organ, limb and bone development in the early stages; in the middle stages, they shift to body length and muscle; and in the later stages, the focus is on fat. The 2.5 and 4.5-year-old yaks in this study were mainly focused on muscle development and fat deposition, which resulted in a phenotype that appeared to be the opposite of acclimatization to a low-oxygen environment. However, yak lungs have developed a relatively well-developed microstructure at these two ages and do not require a large lung weight ratio of yaks to combat the hypoxic environment.

The pulmonary arteries of yaks possess strong vasodilatory and vasoconstrictive capacities when the animals adapt to a higher cardiac output and avoid pulmonary hypertension during adaptation to a low-oxygen environment at a high altitude [25,26]. In the present study, we found that the thickness of pulmonary arteries in yaks increased with age. Previous studies have found that hypoxia disrupts endothelial cell integrity and triggers growth factor influx, leading to smooth muscle cell proliferation and pulmonary artery thickening [27], and vascular remodeling is also promoted in hypoxia [28]. Meanwhile, thicker pulmonary arteries contain smoother muscle and elastic fibers. However, they can accommodate more blood through vasodilatation as the proper ventricle contracts to deliver blood to the whole lung through vasoconstriction. The thickness of the pulmonary arteries gradually increases with increasing body weight during the development of yaks to better supply oxygen to the whole body, which is consistent with the results in the study about Tibetan sheep [5]. In conclusion, the gradual thickening of the pulmonary arteries with age allows yaks to control blood flow in the lungs under hypoxic conditions, thus avoiding pulmonary hypertension.

A higher alveolar surface area, better alveoli diastolic capacity, and the adaptation of yaks to a low-oxygen environment are closely related. In this study, the number of alveoli gradually decreased with age, while the mean area of individual alveoli gradually increased, and the elastic fiber content gradually increased under the same magnification field of view. Alveolar counts were higher in guinea pigs [29] and dogs [30] at high altitudes, whereas Andean geese [7] and yaks [31] had larger alveolar surface areas. This suggests that the increase in the gas exchange surface area in high-altitude animals compensates for the limited availability of oxygen in the high-altitude environment. Calves rely on a large number of alveoli in the same area to increase the surface area for gas exchange, while in youth and adulthood, they rely on the greater surface area of individual alveoli to aid gas exchange. At the same time, the increased elastic fiber content helps the alveoli to expand fully during inspiration and retract during expiration, thus avoiding emphysema. These changes are the same as those exhibited by other highland animals against hypoxic environments, allowing yaks to gradually adapt as they grow from calves to adult yaks.

### 3.2. Gene Expression

Higher pulmonary ventilation and arterial vasodilatory or contractile responses can help exchange oxygen and require larger vascular volumes. The present study found that the mean cross-sectional area of pulmonary micro-arterioles gradually increased with age. The increased cross-sectional area of pulmonary micro-arterioles and a high degree of vascularization of the lungs have been found in studies in Tibetan sheep and Andean geese [5,7]. These morphological changes not only increase the surface area for gas exchange but also increase the vascular volume. Greater blood volume reduces peripheral resistance and avoids pulmonary hypertension. The thickened pulmonary arteries transport blood throughout the lungs by elastic recoil for adequate oxygen exchange, providing abundant oxygen to the organism through higher pulmonary ventilation. These morphological changes are gradually revealed during yak development, which may underlie the adaptation of yaks to hypoxic environments.

Transcriptomics and untargeted metabolomics were applied to analyze further the mechanisms underlying lung tissue cells’ differential response to age. In this study, some of the hub genes belonging to the MEmagenta module (*MYC*, *EREG*, *SOCS3*, *HBEGF*, *ZFP36*) were significantly up-regulated with age. *MYC* and *EREG* are associated with angiogenesis; *MYC* binds to the Vascular Endothelial Growth Factor A (*VEGFA*) promoter and promotes *VEGFA* production and subsequent angiogenesis [32], *EREG* regulates angiogenesis and vascular remodeling as well as stimulating cell proliferation [33], and *HBEGF* is closely associated with the positive regulation of vascular-related smooth muscle cell proliferation [34]. Therefore, it is hypothesized that the increased expression of these three genes with age is one of the reasons for the thickening of the pulmonary arteries with age and the increase in elastic fibers in the pulmonary arteries with age during the gradual development of yaks adapted to the hypoxic environment. When studying the role of *SOCS3* in the regulation of mouse fetal hepatic erythropoiesis, it was found that *SOCS3* plays a crucial role in negatively regulating embryonic hepatic hematopoiesis [35], *HIF-1α* can be stably expressed when the organism is in hypoxic conditions, which in turn regulates a series of genes to adapt to the hypoxic environment [36], and silencing *ZFP36* expression enhances the hypoxia-induced increase in *HIF-1α* protein levels [37]. The expression of *SOCS3* and *ZFP36* gradually increases with age, and generally, it seems that this situation indicates that yaks are becoming less capable of adapting to hypoxia. However, as yaks gradually develop in hypoxic environments, their lungs develop a more refined microstructure and gradually adapt to the hypoxic environment. Thus, there is no need to produce more red blood cells and key proteins capable of responding to hypoxic levels to combat the hypoxic environment, which further suggests that yaks have adapted to the local hypoxic environment.

In addition, *EPHA2* and *TNF* also play a role in angiogenesis. *EPHA2* was found to promote the formation of vasculogenic mimicry (VM) in colorectal cancer (CRC) via PI3K/AKT/mTOR and ERK1/2 signaling [38], and *TNF* plays a role in angiogenesis by synergistically inducing *VEGF* production with IL-1beta (*IL1B*). Whether interleukin-6 (*IL6*) has a role needs to be investigated further. The expression of the above two genes is relatively high in yaks due to the rapid development of organs during the calving period, and in adulthood, due to the larger size of yaks, the lungs require more powerful blood vessels to support their adequate oxygenation of the organism, which may explain why the expression of *EPHA2* and *TNF* is higher in calves and adults than that in young cows.

### 3.3. Metabolomic Insights

Metabolomics is the study of all metabolites of an organism and is closest to phenotypic analysis [39]. Most previous studies on hypoxia adaptation have been performed on animals at different altitudes, allowing for analyzing lung differences [40]. However, this doomed the subjects to ingest different levels of nutrients [41]. The relationship between the metabolites of an organism and the level of nutrition it ingests is very close. Thus, in studies of animals living at different altitudes, it is not possible to accurately target metabolites accordingly. In this study, the subjects were fed the same nutrient level of feed for 6 consecutive months under barn feeding. This ensured, as far as possible, that the nutritional level of the feed did not influence the differences in metabolites.

As an intermediate product of purine metabolism, xanthosine may play a role in adapting yaks to hypoxic environments. Purine metabolites have been found to cause the relaxation of vascular smooth muscle and dilation of micro-arterioles to increase blood flow during hypoxia in cardiomyocytes [38]. In this study, xanthosine, a metabolite belonging to the ME 2 module, was positively correlated with several hub genes and was enriched in the purine metabolic pathway. Xanthosine is involved in the synthesis and catabolism of purine nucleotides, and the concentration of adenosine, a purine metabolite, is elevated in the body when it is exposed to hypoxia, which may be one of the reasons for the adaptation to hypoxia in the yak. Moreover, xanthosine is also enriched in the caffeine metabolic pathway, in which theophylline also exerts a diastolic effect on tracheal smooth muscle [42].

In conclusion, yaks have been adapted to the low-oxygen environment through natural selection and artificial domestication for a long period of time. The yak has been adapted to the low-oxygen environment by physiological structures and gene expression at various stages of growth, but the underlying molecular mechanisms still need to be explored.

## 4. Materials and Methods

### 4.1. Sample Collect

In this study, calves (0.5 years old, *n* = 5, A), young yaks (2.5 years old, *n* = 5) and adult yaks (4.5 years old, *n* = 5) (all bulls in excellent body condition and health) at the same feeding level (Table 3) in Tianzhu Tibetan Autonomous County, Gansu Province (altitude 2800 m) were selected. Live cattle were weighed and then slaughtered, and the lung wet weights were recorded. About 3 g of parenchyma of the middle segment of the lower lobe of the right lung was quickly taken in a cryopreservation tube (Servicebio, Wuhan, China). It was preserved in liquid nitrogen for transcriptomics and metabolomics. Another 1×1×1 cm of right lung lower lobe middle segment parenchyma was taken and preserved in 4% paraformaldehyde (Servicebio, Wuhan, China) for hematoxylin–eosin (HE) (Servicebio, Wuhan, China) and Weigert resorcinol magenta (WRM) (Servicebio, Wuhan, China) staining.

### 4.2. HE and WRM Staining and Analysis

HE and WRM staining were used for alveolar number and area measurements, pulmonary micro-arterial area, micro-arterial wall thickness measurements, and elastic fiber content. In this study, HE and WRM staining was performed on 15 samples; samples were fixed with 4% paraformaldehyde, dehydrated in graded ethanol (SCRC, Shanghai, China) (75%→85%→95%→100%), rinsed in xylene (SCRC, Shanghai, China), and embedded in paraffin wax (Servicebio, Wuhan, China); then, embedded samples were cut into 5 μm thick sections and stained with hematoxylin–eosin (HE) and Weigert resorcinol magenta (WRM), respectively. After scanning, CaseViewer (V2.4.0.119028, 3DHISTECH, Budapest, Hungary) was used to observe the HE sections and calculate the number and area of alveoli, the area of pulmonary micro-arterioles, and the thickness of micro-arteriolar walls. Image-Pro Plus 6.0 (V6.0.0.260, Media Cybernetics, Rockville, MD, USA) was used to observe the WRM sections, and the area of elastin fibers in the lungs was determined to ascertain their content.

### 4.3. Transcriptomic Sequencing and Analysis of Lung Tissue

Transcriptomic analysis was performed on 15 lung tissue samples. RNA was extracted from lung tissues using TRIzol reagent (Thermo Fisher Scientific, Waltham, MA, USA). To ensure the concentration and quality of RNA, the absorption optical density ratio of total RNA was measured using a NanoDrop spectrophotometer (NC2000, Thermo Fisher Scientific, MA, USA), and the integrity of total RNA was examined by RNA-specific agarose electrophoresis. Sequencing libraries were generated according to the following steps. Firstly, mRNA was purified from total RNA using poly-T oligo-attached magnetic beads (Thermo Fisher Scientific, Waltham, MA, USA). Fragmentation was carried out using divalent cations under elevated temperature in an Illumina proprietary fragmentation buffer (Illumina, San Diego, CA, USA). First-strand cDNA was synthesized using random oligonucleotides (Thermo Fisher Scientific, Waltham, MA, USA) and Super Script II (Thermo Fisher Scientific, Waltham, MA, USA). Second-strand cDNA synthesis was subsequently performed using DNA Polymerase I (Thermo Fisher Scientific, Waltham, MA, USA) and RNase H (Thermo Fisher Scientific, Waltham, MA, USA). The remaining overhangs were converted into blunt ends via exonuclease/polymerase activities, and the enzymes were removed. After adenylation of the 3′ends of the DNA fragments, Illumina PE adapter oligonucleotides were ligated to prepare for hybridization. To select cDNA fragments of the preferred 400–500 bp in length, the library fragments were purified using the AMPure XP system (Beckman Coulter, Beverly, CA, USA). DNA fragments with ligated adaptor molecules on both ends were selectively enriched using Illumina PCR Primer Cocktail (Illumina, San Diego, CA, USA) in a 15-cycle PCR reaction. Products were purified (AMPure XP system) and quantified using the Agilent high-sensitivity DNA assay (Agilent Technologies Inc., Santa Clara, CA, USA) on a Bioanalyzer 2100 system (Agilent Technologies Inc., Santa Clara, CA, USA). The sequencing library was then sequenced on the NovaSeq 6000 platform (Illumina, San Diego, CA, USA) of Shanghai Personal Biotechnology Cp. Ltd. to obtain downstream data in FASTQ format.

The raw data were further filtered to obtain clean data. Indexes were constructed using HISAT2 (https://ccb.jhu.edu/software/hisat2/index.shtml, accessed on 12 November 2023), and paired-end clean reads were compared with the reference genome using HISAT2 (https://asia.ensembl.org/Bos_grunniens/Info/Annotation, accessed on 12 November 2023). HTSeq (v0.9.1) was used to statistically compare the Read Count value on each gene as the raw expression of the gene. In order to make the gene expression levels comparable among different genes and samples, FPKM (Fragments Per Kilo bases per Million fragments) was used to normalize the expression amount. GO enrichment analysis was performed using topGO (v2.50.0) to find out the GO terms that were significantly enriched by differential genes, and KEGG pathway enrichment analysis was performed using clusterProfiler (v4.6.0) software to find out the significantly enriched pathways of differential genes.

### 4.4. Metabolomic of Lung Tissue

Metabolites were determined in 15 lung tissues using metabolomics techniques. The lung tissue samples (25 mg ± 1 mg) were taken and mixed with beads and 500 μL of extraction solution (MeOH:ACN:H_2_O, 2:2:1 (*v*/*v*)). The extraction solution contained deuterated internal standards. The mixed solution was vortexed for 30 s. Then, the mixed samples were homogenized (35 Hz, 4 min) and sonicated for 5 min in 4 °C water bath; this step was repeated three times. The samples were incubated for 1 h at −40 °C to precipitate proteins. Then, the samples were centrifuged at 12,000 rpm (RCF = 13,800 (× *g*), R = 8.6 cm) for 15 min at 4 °C. The supernatant was transferred to a fresh glass vial for analysis. The quality control (QC) sample was prepared by mixing an equal aliquot of the supernatant of samples.

For polar metabolites, LC-MS/MS analyses were performed using an UHPLC system (Thermo Fisher Scientific, Waltham, MA, USA) with a Waters ACQUITY UPLC BEH Amide (2.1 mm × 50 mm,1.7 μm) coupled to an Orbitrap Exploris 120 mass spectrometer (Thermo Fisher Scientific, Waltham, MA, USA). The mobile phase consisted of 25 mmol/L ammonium acetate (Merck, Darmstadt, German) and 25 ammonia hydroxide (Merck, Darmstadt, German) in water (pH = 9.75) (A) and acetonitrile (Merck, Darmstadt, German) (B). The auto-sampler temperature was 4 °C, and the injection volume was 2 μL. The Orbitrap Exploris 120 mass spectrometer was used for its ability to acquire MS/MS spectra on information-dependent acquisition (IDA) mode in the control of the acquisition software (Xcalibur 4.3, Thermo Fisher Scientific, Waltham, MA, USA). In this mode, the acquisition software continuously evaluates the full-scan MS spectrum. The ESI source conditions were set as follows: sheath gas flow rate as 50 Arb, Aux gas flow rate as 15 Arb, capillary temperature 320 °C, full MS resolution as 60,000, MS/MS resolution as 15,000, collision energy SNCE 20/30/40, spray voltage as 3.8 kV (positive) or −3.4 kV (negative), respectively.

To process the raw data collected using MassLynx (V4.2, Waters Corporation, Milford, MA, USA), Progenesis QI (V4.0, Waters Corporation, Milford, MA, USA) software was utilized for operations such as peak extraction and peak pairing. Identification of the compounds was conducted based on the online METLIN database (http://metlin.scripps.edu, accessed on 27 December 2023)of Progenesis QI (V4.0, Waters Corporation, Milford, MA, USA) software and Biomark’s self-constructed libraries.

### 4.5. RT-qPCR Validation of RNA-Seq Results

RT-qPCR was performed on eleven selected genes to validate the gene expression results obtained by RNA-seq. PCR primers of these genes were designed using primer (V5.0, Premier Biosoft, San Francisco, CA, USA) and synthesized by the Takara Biotech Co., Ltd. (Dalian, China) (Table 4). RNA samples were extracted in the same way as for RNA-seq analysis. The RNA samples used for RNA-seq are also used to synthesize cDNA using the SuperScript TM II reverse transcriptase (Invitrogen, Carlsbad, CA, USA), and RT-qPCR was performed in four replicates using a 2×ChamQ SYBR qPCR Master (Vazyme, Nanjing, China) on an Applied Biosystems QuantStudio^®^ six Flex (Thermo Lifetech, Waltham, CA, USA) with β-actin as the internal reference gene and the 2^−ΔΔCt^ method to calculate the relative gene expression.

### 4.6. Statistical Analysis

One-way ANOVA in SPSS software (version 25, SPSS Inc., Chicago, IL, USA) was applied to compare the difference between the indicators, including lung tissue section parameters and DGEs, among the three groups. The results with *p* < 0.05 were adjudicated as statistical significance.

The weighted gene co-expression network analysis (WGCNA) algorithm [43] in the R package was used to access the modules of genes and metabolites related to the characterization of lung tissue development. After selecting the target module, the genes with |GS| > 0.2 (GS, Gene Significance) and |MM| > 0.8 (MM, module membership) were screened as hub genes.

The functional enrichment analysis of metabolites was performed using the MetaboAnalyst 5.0 platform (www.metaboanalyst.ca, accessed on 14 January 2024).

## 5. Conclusions

**Figure 9:**
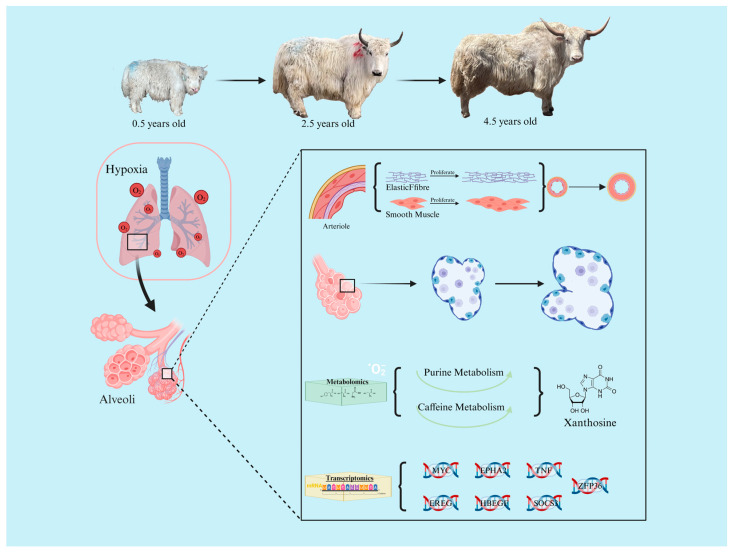
Microscopic changes in yak lung adaptation to low-oxygen environment, important genes and metabolites.

The present study demonstrates that yaks adapt to the hypoxic environment by increasing the pulmonary artery volume and thickness, alveolar area, and elastic fiber content during development. In addition to this, some genes related to angiogenesis (*MYC*, *EPHA2*, *TNF*), fiber formation (*EREG*), smooth muscle proliferation (*HBEGF*), erythropoiesis (*SOCS3*), and hypoxia response (*ZFP36*), as well as metabolites enriched in the purine metabolism pathway and caffeine metabolism pathway, which were identified by the transcriptomics data, further validated these findings (Figure 9). The data from this study provide important insights into yak acclimatization to high altitudes. Identifying genes required for natural acclimatization to high altitude may help improve the treatment, understanding, and prevention of altitude sickness and other hypoxia-related diseases in humans.

## Figures and Tables

**Table 1:** Histomorphology of lungs of yaks of different ages.

Lung Morphology	Groups			SEM	*p*-Value
	0.5 Years Old	2.5 Years Old	4.5 Years Old		
Alveolar number	563.60 ^a^	449.80 ^b^	403.20 ^c^	19.46	<0.01
Average alveolar area, μm^2^	1305.44 ^b^	1922.81 ^a^	1968.18 ^a^	102.74	<0.01
Percentage of elastic fibers, %	3.20 ^c^	4.04 ^b^	4.48 ^a^	0.15	<0.01
Wall thickness of micro arteries, μm	22.79 ^c^	37.17 ^b^	76.46 ^a^	6.13	<0.01
Micro-arterial area, μm^2^	3697.64 ^c^	4726.65 ^b^	9105.18 ^a^	639.19	<0.01

^a,b,c^ Values in a row with no common superscripts differ significantly (*p <* 0.01).

**Table 2:** Summary of the RNA-seq data.

Sample	Average Raw Reads	Average Clean Reads	Average Useful Reads Rate	Average Mapped Reads	Average Multiple Mapped	Average Uniquely Mapped
A	44,770,822	44,258,157	98.88%	41,661,962 (94.13%)	3,845,957 (9.22%)	37,816,005 (90.78%)
B	45,910,218	45,422,947	98.94%	42,744,048 (94.10%)	3,975,168 (9.28%)	38,768,880 (90.72%)
C	43,345,683	42,860,926	98.88%	40,212,112 (93.81%)	3,706,944 (9.21%)	36,505,168 (90.79%)

**Table 3:** Dietary combinations and nutritional levels (dry matter basis).

Item		Item	
Ingredient		Nutrient levels	
Corn stover, %	38.90	CNE (MJ/kg)	81.54
Oat grass, %	13.30	DM, %	92.97
Scutellaria baicalensis stalks, %	12.70	CP, %	13.43
Corn, %	14.90	EE, %	8.95
Bran, %	7.20	NDF, %	43.42
Rapeseed meal, %	6.80	ADF, %	21.36
Soy protein powder, %	3.60	Ca, %	0.52
Salt, %	0.60	P, %	0.34
Premix, %	1.90		
Total, %	100.00		

**Table 4:** Primers of RT-qPCR.

Name	Forward (5′ → 3′)	Reverse (5′ → 3′)
*ARL5C*	GGAACGGGAGCACAAGGTTA	CCCACATGAGGAAGTGGGTC
*HK3*	CTGCCGTGGTGGAGAAGATT	GAGCTGGTTGCTGGAGACTT
*PER1*	GCTCCTCCAGTGACAGCAAT	GGGCCCGGGACTCAATAAAA
*MED11*	CGGCTCAGATCCGCTATCTC	CGAGCATAGTCCACTCGCTT
*DNAH7*	CACCAGATTAGCTGCCCACA	TGGTTTTATCCCGTTGGCGA
*FOS*	ACTACGAGGCATCCTCCTCC	CCAGATCGGTGCAGTAGTCC
*KLF11*	GTTGTAGAGACTGCACCCCC	TTGAAGGGCAGAGCGACATT
*NOTCH4*	CCTCCCATTTCTGCCACTGT	TGACATGCGTCTGGTTCCTC
*β-actin*	GACCTCTACGCCAACACG	CTGGAAGGTGGACAGCGAG

## Data Availability

The RNA-seq data provided in this study can be found in the GenBank Sequence Read Archive (SRA) database under accession number PRJNA1164457 (https://submit.ncbi.nlm.nih.gov/subs/sra/, 24 September 2024).

## References

[1] Thompson L.G., Yao T., Mosley-Thompson E., Davis M.E., Henderson K.A., Lin P.-N. (2000). A High-Resolution Millennial Record of the South Asian Monsoon from Himalayan Ice Cores. Science.

[2] Beall C.M. (2007). Two routes to functional adaptation: Tibetan and Andean high-altitude natives. Proc. Natl. Acad. Sci. USA.

[3] Jin Y.C., Yan Z.X., Li S.S., Liu L.J. (2017). Research progress on male sterility of cattle-Yak in China. Chin. Qinghai J. Anim. Vet. Sci..

[4] Zhao P., Li S., He Z., Zhao F., Wang J., Liu X., Li M., Hu J., Zhao Z., Luo Y. (2022). Physiology and Proteomic Basis of Lung Adaptation to High-Altitude Hypoxia in Tibetan Sheep. Animals.

[5] Zhao P., Zhao F., Hu J., Wang J., Liu X., Zhao Z., Xi Q., Sun H., Li S., Luo Y. (2022). Physiology and Transcriptomics Analysis Reveal the Contribution of Lungs on High-Altitude Hypoxia Adaptation in Tibetan Sheep. Front. Physiol..

[6] Howell K., Preston R.J., McLoughlin P. (2002). Chronic hypoxia causes angiogenesis in addition to remodelling in the adult rat pulmonary circulation. J. Physiol..

[7] Maina J.N., McCracken K.G., Chua B., York J.M., Milsom W.K. (2017). Morphological and morphometric specializations of the lung of the Andean goose, Chloephaga melanoptera: A lifelong high-altitude resident. PLoS ONE.

[8] Scott G.R., Elogio T.S., Lui M.A., Storz J.F., Cheviron Z.A. (2015). Adaptive Modifications of Muscle Phenotype in High-Altitude Deer Mice Are Associated with Evolved Changes in Gene Regulation. Mol. Biol. Evol..

[9] Tate K.B., Wearing O.H., Ivy C.M., Cheviron Z.A., Storz J.F., McClelland G.B., Scott G.R. (2020). Coordinated changes across the O_2_ transport pathway underlie adaptive increases in thermogenic capacity in high-altitude deer mice. Proc. R. Soc. B Biol. Sci..

[10] Kong X., Dong X., Yang S., Qian J., Yang J., Jiang Q., Li X., Wang B., Yan D., Lu S. (2019). Natural selection on TMPRSS6 associated with the blunted erythropoiesis and improved blood viscosity in Tibetan pigs. Comp. Biochem. Physiol. Part B Biochem. Mol. Biol..

[11] Gou X., Wang Z., Li N., Qiu F., Xu Z., Yan D., Yang S., Jia J., Kong X., Wei Z. (2014). Whole-genome sequencing of six dog breeds from continuous altitudes reveals adaptation to high-altitude hypoxia. Genome Res..

[12] Xin J.-W., Chai Z.-X., Zhang C.-F., Zhang Q., Zhu Y., Cao H.-W., Ji Q.-M., Zhong J.-C. (2019). Transcriptome profiles revealed the mechanisms underlying the adaptation of yak to high-altitude environments. Sci. Rep..

[13] Zhang Y., Zhou M., Liang Y., Li R., Zhang L., Chen S., Yang K., Ding H., Tan X., Zhang Q. (2023). Study of Transcriptomic Analysis of Yak (*Bos grunniens*) and Cattle (*Bos taurus*) Pulmonary Artery Smooth Muscle Cells under Oxygen Concentration Gradients and Differences in Their Lung Histology and Expression of Pyruvate Dehydrogenase Kinase 1-Related Factors. Animals.

[14] Li J., Meng X., Wang L., Yu Y., Yu H., Wei Q. (2021). Changes in the expression levels of elastic fibres in yak lungs at different growth stages. BMC Dev. Biol..

[15] Qi X., Zhang Q., He Y., Yang L., Zhang X., Shi P., Yang L., Liu Z., Zhang F., Liu F. (2018). The transcriptomic landscape of yaks reveals molecular pathways for high altitude adaptation. Genome Biol. Evol..

[16] Li P., Du L., Li W., Fan Z., Zeng D., Chen H., Zhou L., Yi Y., Yang N., Dou K. (2017). Generation and characterization of the blood transcriptome of Macaca thibetana and comparative analysis with M. mulatta. Mol. Biosyst..

[17] Jia C., Kong X., Koltes J.E., Gou X., Yang S., Yan D., Lu S., Wei Z. (2016). Gene Co-Expression Network Analysis Unraveling Transcriptional Regulation of High-Altitude Adaptation of Tibetan Pig. PLoS ONE.

[18] Zhang B., Qiangba Y., Shang P., Wang Z., Ma J., Wang L., Zhang H. (2015). A Comprehensive MicroRNA Expression Profile Related to Hypoxia Adaptation in the Tibetan Pig. PLOS ONE.

[19] Sato A., Ishigami A. (2023). Effects of heated tobacco product aerosol extracts on DNA methylation and gene transcription in lung epithelial cells. Toxicol. Appl. Pharmacol..

[20] Jin H., Chang S.-H., Xu C.-X., Shin J.-Y., Chung Y.-S., Park S.-J., Lee Y.-S., An G.-H., Lee K.-H., Cho M.-H. (2007). High Dietary Inorganic Phosphate Affects Lung through Altering Protein Translation, Cell Cycle, and Angiogenesis in Developing Mice. Toxicol. Sci..

[21] Paul S., Gangwar A., Arya A., Bhargava K., Ahmad Y. (2021). Modulation of lung cytoskeletal remodeling, RXR based metabolic cascades and inflammation to achieve redox homeostasis during extended exposures to lowered pO2. Apoptosis.

[22] Sanders C.L., Adee R.R. (1968). Phagocytosis of Inhaled Plutonium Oxide-^239^ Pu Particles by Pulmonary Macrophages. Science.

[23] Kiyamu M., Bigham A., Parra E., León-Velarde F., Rivera-Chira M., Brutsaert T.D. (2012). Developmental and genetic components explain enhanced pulmonary volumes of female peruvian quechua. Am. J. Phys. Anthr..

[24] Yang Y., Gao C., Yang T., Sha Y., Cai Y., Wang X., Yang Q., Liu C., Wang B., Zhao S. (2021). Characteristics of Tibetan pig lung tissue in response to a hypoxic environment on the Qinghai–Tibet Plateau. Arch. Anim. Breed..

[25] Heath D., Williams D., Dickinson J. (1984). The pulmonary arteries of the yak. Cardiovasc. Res..

[26] Durmowicz A.G., Hofmeister S., Kadyraliev T.K., Aldashev A.A., Stenmark K.R. (1993). Functional and structural adaptation of the yak pulmonary circulation to residence at high altitude. J. Appl. Physiol..

[27] Wang D., Zhang H., Li M., Frid M.G., Flockton A.R., McKeon B.A., Yeager M.E., Fini M.A., Morrell N.W., Pullamsetti S.S. (2014). MicroRNA-124 Controls the Proliferative, Migratory, and Inflammatory Phenotype of Pulmonary Vascular Fibroblasts. Circ. Res..

[28] Maron B.A., Oldham W.M., Chan S.Y., Vargas S.O., Arons E., Zhang Y.-Y., Loscalzo J., Leopold J.A. (2014). Upregulation of Steroidogenic Acute Regulatory Protein by Hypoxia Stimulates Aldosterone Synthesis in Pulmonary Artery Endothelial Cells to Promote Pulmonary Vascular Fibrosis. Circulation.

[29] Hsia C.C., Carbayo J.J.P., Yan X., Bellotto D.J. (2005). Enhanced alveolar growth and remodeling in Guinea pigs raised at high altitude. Respir. Physiol. Neurobiol..

[30] Hsia C.C.W., Jr R.L.J., McDonough P., Dane D.M., Hurst M.D., Fehmel J.L., Wagner H.E., Wagner P.D. (2007). Residence at 3,800-m altitude for 5 mo in growing dogs enhances lung diffusing capacity for oxygen that persists at least 2.5 years. J. Appl. Physiol..

[31] Ding X., Liang C., Guo X., Wu X., Wang H., Johnson K., Yan P. (2014). Physiological insight into the high-altitude adaptations in domesticated yaks (Bos grunniens) along the Qinghai-Tibetan Plateau altitudinal gradient. Livest. Sci..

[32] Shi Y., Xu X., Zhang Q., Fu G., Mo Z., Wang G.S., Kishi S., Yang X.-L., States U. (2014). tRNA synthetase counteracts c-Myc to develop functional vasculature. eLife.

[33] Riese D.J., Cullum R.L. (2014). Epiregulin: Roles in normal physiology and cancer. Semin. Cell Dev. Biol..

[34] Wang Y., Xian H. (2022). Identifying Genes Related to Acute Myocardial Infarction Based on Network Control Capability. Genes.

[35] Marine J.-C., McKay C., Wang D., Topham D.J., Parganas E., Nakajima H., Pendeville H., Yasukawa H., Sasaki A., Yoshimura A. (1999). SOCS3 Is Essential in the Regulation of Fetal Liver Erythropoiesis. Cell.

[36] Groenman F., Rutter M., Caniggia I., Tibboel D., Post M. (2006). Hypoxia-inducible Factors in the First Trimester Human Lung. J. Histochem. Cytochem..

[37] Chamboredon S., Ciais D., Desroches-Castan A., Savi P., Bono F., Feige J.-J., Cherradi N. (2011). Hypoxia-inducible factor-1α mRNA: A new target for destabilization by tristetraprolin in endothelial cells. Mol. Biol. Cell.

[38] Aldian D., Harisa L.D., Mitsuishi H., Tian K., Iwasawa A., Yayota M. (2023). Diverse Forage Improves Lipid Metabolism and Antioxidant Capacity in Goats, As Revealed by Metabolomics. Animal.

[39] Ge Q., Guo Y., Zheng W., Zhao S., Cai Y., Qi X. (2021). Molecular mechanisms detected in yak lung tissue via transcriptome-wide analysis provide insights into adaptation to high altitudes. Sci. Rep..

[40] Ayres J.S. (2020). A metabolic handbook for the COVID-19 pandemic. Nat. Metab..

[41] Merin R.G. (1992). Physiology, pathophysiology and pharmacology of the coronary circulation with particular emphasis on anes-thetics. Anaesthesiol. Reanim..

[42] Ingvast-Larsson C. (1991). Relaxant effects of theophylline and clenbuterol on tracheal smooth muscle from horse and rat in vitro. J. Veter-Pharmacol. Ther..

[43] Langfelder P., Horvath S. (2008). WGCNA: An R package for weighted correlation network analysis. BMC Bioinform..

